# Supplementary material for: Characterization of Two New Shiga Toxin-Producing Escherichia coli O103-Infecting Phages Isolated from an Organic Farm
Source: Microorganisms. 2021 Jul 17;9(7):1527. doi: 10.3390/microorganisms9071527 (PMC8303462; doi:10.3390/microorganisms9071527)
Supplement: Supplementary file 1 [file microorganisms-09-01527-s001.zip › microorganisms-1280391-supplementary.pdf]

microorganisms

MDPI

Supplementary Materials

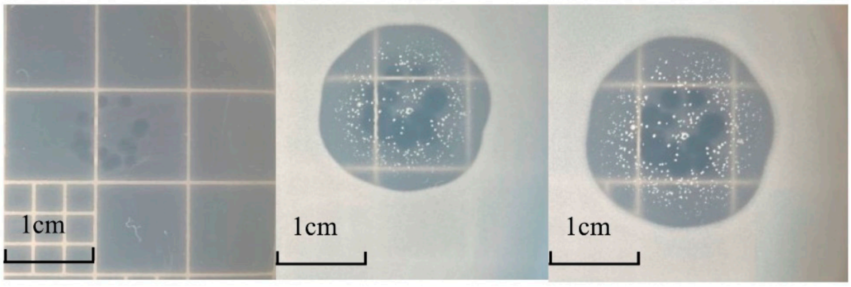

(a) (b) (c)

Figure S1. The appearance of plaques formed by phage Ro103C3lw on *E. coli* O103:H2 strain RM10744. The haloes of the lysis zones (plaques) grew in size from 0.72 cm to 2.15 cm after 3 h (a), 23 h (b), 27 h (c) of incubation at 37°C.

Table S1. List of annotated CDSs with the size, location, and predicted functions in the genome of Ro103C3lw.

No.	Predicted CDSs*	Start (bp)	Stop (bp)	Length (bp)	Direction	Functional Category [a]
1	hypothetical protein	837	995	159	forward	n/a
2	hypothetical protein	1166	1498	333	forward	n/a
3	hypothetical protein	1498	1653	156	forward	n/a
4	hypothetical protein	1656	1781	126	forward	n/a
5	hypothetical protein	1941	2138	198	forward	n/a
6	hypothetical protein	2138	2464	327	forward	n/a
7	DNA-directed RNA polymerase	2561	5227	2667	forward	DNA regulation and replication
8	hypothetical protein	5241	5441	201	forward	n/a
9	hypothetical protein	5521	5976	456	forward	n/a
10	hypothetical protein	6063	6242	180	forward	n/a
11	hypothetical protein	6247	6510	264	forward	n/a
12	DNA ligase	6510	7571	1062	forward	DNA regulation and replication
13	hypothetical protein	7678	7932	255	forward	n/a
14	hypothetical protein	7932	8309	378	forward	n/a
15	putative PTS sucrose transporter subunit IIABC	8388	8564	177	forward	DNA regulation and replication
16	host RNA polymerase inhibitor	8545	8715	171	forward	DNA regulation and replication

17	helix-destabilizing protein	8759	9457	699	forward	DNA regulation and replication
18	endonuclease	9538	9912	375	forward	DNA packaging
19	hypothetical protein	9909	10133	225	forward	n/a
20	endolysin	10123	10581	459	forward	Host cell lysis
21	hypothetical protein	10596	10808	213	forward	n/a
22	hypothetical protein	10881	10976	96	forward	n/a
23	primase/helicase protein	10987	12690	1704	forward	DNA regulation and replication
24	hypothetical protein	12735	12899	165	forward	n/a
25	DNA-directed DNA polymerase	12973	15144	2172	forward	DNA regulation and replication
26	putative HNS binding protein	15144	15428	285	forward	DNA regulation and replication
27	hypothetical protein	15425	15634	210	forward	n/a
28	hypothetical protein	15631	15906	276	forward	n/a
29	hypothetical protein	15899	16207	309	forward	n/a
30	exonuclease	16200	17066	867	forward	DNA packaging
31	hypothetical protein	17274	17546	273	forward	n/a
32	hypothetical protein	17558	17782	225	forward	n/a
33	hypothetical protein	17823	18188	366	forward	n/a
34	head-to-tail joining protein	18447	20015	1569	forward	Phage morphogenesis
35	capsid assembly protein	20113	20988	876	forward	Phage morphogenesis
36	minor capsid protein	21115	22164	1050	forward	Phage morphogenesis
37	hypothetical protein	22233	22427	195	forward	n/a
38	putative tail fiber protein /tail tubular protein A	22488	23054	567	forward	Phage morphogenesis
39	putative tail fiber protein	23066	25432	2367	forward	Phage morphogenesis
40	putative internal (core) protein	25519	25980	462	forward	Phage morphogenesis
41	putative internal virion protein	25965	26552	588	forward	Phage morphogenesis
42	internal virion protein	26564	28846	2283	forward	Phage morphogenesis

43	hypothetical protein	28849	29298	450	forward	n/a
44	internal virion protein D	29280	33176	3897	forward	Phage morphogenesis
45	putative tail fiber protein	33241	35331	2091	forward	Phage morphogenesis
46	holin	35372	35566	195	forward	Host cell lysis
47	putative DNA packaging protein small subunit	35563	35826	264	forward	DNA packaging
48	hypothetical protein	35883	36254	372	forward	n/a
49	endopeptidase Rz	36273	36722	450	forward	Host cell lysis
50	terminase large subunit	36719	38482	1764	forward	DNA packaging
51	hypothetical protein	38771	38929	159	forward	n/a

*CDS means coding DNA sequence.

[a] n/a means the CDSs are not annotated with any predicted functions.

Table S2. List of annotated CDSs with the size, location, and predicted functions in the genome of Pr103Blw.

No.	Predicated CDSs*	Start (bp)	Stop (bp)	Length (bp)	Direction	Functional category [a]
1	hypothetical protein	1213	1761	549	reverse	n/a
2	hypothetical protein	1775	2239	465	reverse	n/a
3	hypothetical protein	2229	2657	429	reverse	n/a
4	hypothetical protein	2712	2936	225	reverse	n/a
5	hypothetical protein	3013	3516	504	reverse	n/a
6	hypothetical protein	3513	3731	219	reverse	n/a
7	hypothetical protein	3815	4396	582	reverse	n/a
8	hypothetical protein	4396	4740	345	reverse	n/a
9	hypothetical protein	4733	5026	294	reverse	n/a
10	hypothetical protein	5026	5424	399	reverse	n/a
11	hypothetical protein	5417	5815	399	reverse	n/a
12	Putative lysis protein	5866	6330	465	reverse	Host cell lysis
13	Putative tail protein	6330	7466	1137	reverse	Phage morphogenesis
14	Tail protein	7463	7834	372	reverse	Phage morphogenesis
15	hypothetical protein	7883	8473	591	reverse	n/a
16	hypotherial protein	8467	8700	234	reverse	n/a
17	hypothetical protein	9093	9464	372	forward	n/a
18	hypothetical protein	9546	10961	1416	forward	n/a
19	tRNA-Pro	11128	11207	80	forward	tRNA
20	tRNA-Glu	11215	11292	78	forward	tRNA
21	tRNA-Met	11384	11460	77	forward	tRNA
22	tRNA-Asn	11544	11620	77	forward	tRNA
23	tRNA-Tyr	11629	11716	88	forward	tRNA
24	tRNA-Asp	11722	11798	77	forward	tRNA
25	tRNA-Lys	12229	12304	76	forward	tRNA
26	tRNA-Met	12383	12459	77	forward	tRNA

27	tRNA-Ile	12461	12536	76	forward	tRNA
28	tRNA-Arg	12783	12859	77	forward	tRNA
29	tRNA-Ser	13098	13187	90	forward	tRNA
30	tRNA-Leu	13442	13518	77	forward	tRNA
31	tRNA-Lys	13526	13601	76	forward	tRNA
32	tRNA-Ala	13608	13683	76	forward	tRNA
33	tRNA-Gly	13690	13764	75	forward	tRNA
34	tRNA-Thr	13772	13847	76	forward	tRNA
35	tRNA-Val	13944	14018	75	forward	tRNA
36	tRNA-Leu	14020	14097	78	forward	tRNA
37	tRNA-Arg	14103	14178	76	forward	tRNA
38	hypothetical protein	14196	14438	243	forward	n/a
39	hypothetical protein	14435	14992	558	forward	n/a
40	tRNA-Gln	15001	15076	76	forward	tRNA
41	tRNA-Leu	15079	15157	79	forward	tRNA
42	tRNA-Gln	15163	15238	76	forward	tRNA
43	tRNA-His	15270	15345	76	forward	tRNA
44	tRNA-Phe	15352	15427	76	forward	tRNA
45	hypothetical protein	15417	16178	762	forward	n/a
46	tRNA-Ser	16181	16273	93	forward	tRNA
47	tRNA-Cys	16278	16353	76	forward	tRNA
48	hypothetical protein	16576	16776	201	forward	n/a
49	Terminase, large subunit	16798	18399	1602	forward	DNA packaging
50	hypothetical protein	18416	19882	1467	forward	n/a
51	hypothetical protein	19882	20382	501	forward	n/a
52	hypothetical protein	20382	20714	333	forward	n/a
53	Peptidase_S49 domain-containing protein	20726	22072	1347	forward	Phage morphogenesis
54	hypothetical protein	22084	22461	378	forward	n/a
55	Major capsid protein	22495	23601	1107	forward	Phage morphogenesis
56	hypothetical protein	23623	24072	450	forward	n/a
57	Tail fibers protein	24072	24554	483	forward	Phage morphogenesis
58	hypothetical protein	24551	24952	402	forward	n/a
59	hypothetical protein	24927	25526	600	forward	n/a
60	Tail sheath	25527	26879	1353	forward	Phage morphogenesis
61	hypothetical protein CDS	26895	27341	447	forward	n/a
62	Tape measure chaperone	27414	27812	399	forward	Phage morphogenesis
63	Tape measure chaperone	27815	28054	240	forward	Phage morphogenesis
64	Tail length tape-measure protein	28054	30282	2229	forward	Phage morphogenesis
65	hypothetical protein	30282	31079	798	forward	n/a
66	hypothetical protein	31079	31420	342	forward	n/a
67	hypothetical protein	31420	32397	978	forward	n/a
68	hypothetical protein	32397	33020	624	forward	n/a

69	Baseplate wedge protein	33020	33439	420	forward	Phage morphogenesis
70	hypothetical protein	33439	34908	1470	forward	n/a
71	hypothetical protein	34911	35765	855	forward	n/a
72	hypothetical protein	35765	36067	303	forward	n/a
73	Putative tail fiber protein GP37	36070	37245	1176	forward	Phage morphogenesis
74	Tail fibers protein	37292	39664	2373	forward	Phage morphogenesis
75	hypothetical protein	39744	39941	198	forward	n/a
76	Putative holin	39938	40309	372	forward	Host cell lysis
77	Thymidylate synthase	40347	41246	900	reverse	DNA regulation and replication
78	Dihydrofolate reductase	41248	41793	546	reverse	DNA regulation and replication
79	hypothetical protein	41790	42050	261	reverse	n/a
80	hypothetical protein	42051	42566	516	reverse	n/a
81	hypothetical protein	42580	42939	360	reverse	n/a
82	Putative transcriptional regulatory protein	42941	43240	300	reverse	DNA regulation and replication
83	hypothetical protein	43233	43445	213	reverse	n/a
84	hypothetical protein	43447	43830	384	reverse	n/a
85	DNA ligase	44263	45366	1104	reverse	DNA regulation and replication
86	hypothetical protein	45341	45901	561	reverse	n/a
87	hypothetical protein	45904	46095	192	reverse	n/a
88	hypothetical protein	46088	46255	168	reverse	n/a
89	hypothetical protein	46252	46554	303	reverse	n/a
90	hypothetical protein	46716	46862	147	reverse	n/a
91	hypothetical protein	46859	47077	219	reverse	n/a
92	hypothetical protein	47103	47282	180	reverse	n/a
93	hypothetical protein	47279	47488	210	reverse	n/a
94	Putative DNA polymerase	47550	50270	2721	reverse	DNA regulation and replication
95	hypothetical protein	50507	50929	423	forward	n/a
96	Minor tail protein	50931	51743	813	forward	Phage morphogenesis
97	Putative deoxynucleotide monophosphate kinase	51805	52548	744	forward	DNA regulation and replication
98	hypothetical protein	52563	52757	195	forward	n/a
99	Putative phage DNA primase/helicase	52750	54735	1986	forward	DNA regulation and replication
100	hypothetical protein	54710	54991	282	forward	n/a
101	hypothetical protein	54988	55137	150	forward	n/a
102	hypothetical protein	55210	56067	858	forward	n/a
103	Putative exodeoxyribonuclease	56130	57170	1041	forward	DNA packaging
104	NAD synthetase	57160	57657	498	forward	DNA regulation and replication
105	hypothetical protein	57679	57927	249	forward	n/a

106	Nucleoside triphosphate pyrophosphohydrolase	57903	58658	756	forward	DNA regulation and replication
107	hypothetical protein	58639	58962	324	forward	n/a
108	hypothetical protein	59054	59287	234	forward	n/a
109	Ribonucleoside-diphosphate reductase	59334	61568	2235	forward	DNA regulation and replication
110	hypothetical protein	61540	61881	342	forward	n/a
111	Ribonucleoside diphosphate reductase beta chain	61878	62951	1074	forward	DNA regulation and replication
112	Glutaredoxin domain-containing protein	62951	63193	243	forward	DNA regulation and replication
113	hypothetical protein	63186	63392	207	forward	n/a
114	Anaerobic ribonucleoside-triphosphate reductase	63441	65585	2145	forward	DNA regulation and replication
115	Tail tube protein	65937	66332	396	forward	Phage morphogenesis
116	hypothetical protein	66329	66628	300	forward	n/a
117	Anaerobic NTP reductase	66638	67123	486	forward	DNA regulation and replication
118	hypothetical protein	67086	67463	378	forward	n/a
119	hypothetical protein	67430	67687	258	forward	n/a
120	hypothetical protein	67690	68010	321	forward	n/a
121	hypothetical protein	68095	68577	483	forward	n/a
122	hypothetical protein	68570	68848	279	forward	n/a
123	hypothetical protein	68862	69326	465	forward	n/a
124	hypothetical protein	69360	69812	453	forward	n/a
125	Ribose-phosphate pyrophosphokinase	69803	70729	927	forward	DNA regulation and replication
126	Putative nictotinate phosphoribosyltransferase	70775	72556	1782	forward	DNA regulation and replication
127	hypothetical protein	72610	72954	345	forward	n/a
128	hypothetical protein	72960	73295	336	forward	n/a
129	Putative membrane protein	73277	73453	177	forward	Phage morphogenesis
130	hypothetical protein	73353	73469	117	forward	n/a
131	RIIA lysis inhibitor	73482	75848	2367	forward	DNA regulation and replication
132	RIIB protein	75928	77037	1110	forward	DNA regulation and replication
133	hypothetical protein	77138	77686	549	forward	n/a
134	Polynucleotide kinase	77664	78359	696	forward	DNA regulation and replication
135	hypothetical protein	78371	78835	465	forward	n/a
136	I-spanin	78888	79235	348	forward	Host cell lysis
137	O-spanin	79177	79398	222	forward	Host cell lysis
138	hypothetical protein	79395	79664	270	forward	n/a
139	hypothetical protein	79661	79975	315	forward	n/a

140	Tail assembly protein	79965	80291	327	forward	Phage morphogenesis
141	hypothetical protein	80266	80499	234	forward	n/a
142	hypothetical protein	80492	80692	201	forward	n/a
143	Macro domain-containing protein	80693	81478	786	forward	DNA regulation and replication
144	hypotherical protein	81714	81872	159	reverse	n/a
145	hypothetical protein	82000	82380	381	forward	n/a
146	hypothetical protein	82470	82760	291	forward	n/a
147	Tail sheath monomer	82849	83145	297	forward	Phage morphogenesis
148	hypothetical protein	83139	83471	333	forward	n/a
149	hypothetical protein	83560	83832	273	forward	n/a
150	hypothetical protein	83917	84321	405	forward	n/a
151	Prohead assembly scaffold protein	84425	84628	204	forward	Phage morphogenesis
152	hypothetical protein	84713	85255	543	forward	n/a
153	hypothetical protein	85322	85555	234	forward	n/a
154	hypothetical protein	85647	85988	342	forward	n/a
155	hypothetical protein	86211	86741	531	forward	n/a
156	hypothetical protein	86804	87067	264	forward	n/a
157	hypothetical protein	87135	87383	249	forward	n/a

*CDS means coding DNA sequence.

[a] n/a means the CDSs are not annotated with any predicted functions.
